# NaCl Inhibits Citrinin and Stimulates *Monascus* Pigments and Monacolin K Production

**DOI:** 10.3390/toxins11020118

**Published:** 2019-02-15

**Authors:** Zhixin Zhen, Xiaoqian Xiong, Yingbao Liu, Jialan Zhang, Shaojin Wang, Li Li, Mengxiang Gao

**Affiliations:** College of Life Science, Yangtze University, Jingzhou 434025, Hubei, China; zhenzhixin@yangtzeu.edu.cn (Z.Z.); 201672408@yangtzeu.edu.cn (X.X.); liuyingbao@yangtzeu.edu.cn (Y.L.); zhangjl@yangtzeu.edu.cn (J.Z.); shaojinwang@nwafu.edu.cn (S.W.); lily2012@yangtzeu.edu.cn (L.L.)

**Keywords:** citrinin, *Monascus purpureus*, secondary metabolites, growth, antioxidant

## Abstract

Applications of beneficial secondary metabolites produced by *Monascus purpureus* (*M. purpureus*) could be greatly limited for citrinin, a kidney toxin. The link of NaCl with cell growth and secondary metabolites in *M. purpureus* was analyzed with supplementations of different concentrations of NaCl in medium. The content of citrinin was reduced by 48.0% but the yellow, orange, red pigments and monacolin K productions were enhanced by 1.7, 1.4, 1.4 and 1.4 times, respectively, compared with those in the control using NaCl at 0.02 M at the 10th day of cultivation. NaCl didn’t affect the cell growth of *M. purpureus*. This was verified through the transcriptional up-regulation of citrinin synthesis genes (*pksCT* and *ctnA*) and the down-regulation of the *Monascus* pigments (MPs) synthesis genes (*pksPT* and *pigR*). Moreover, the reactive oxygen species (ROS) levels were promoted by NaCl at the 2nd day of cultivation, and then inhibited remarkably with the extension of fermentation time. Meanwhile, the activities of superoxide dismutase (SOD) and catalase (CAT), and the contents of total glutathione (T-GSH) were significantly enhanced in the middle and late stages of cultivation. The inhibition effect on colony size and the growth of aerial mycelia was more obvious with an increased NaCl concentration. Acid and alkaline phosphatase (ACP and AKP) activities dramatically increased in NaCl treatments. NaCl could participate in secondary metabolites synthesis and cell growth in *M. purpureus*.

## 1. Introduction

*Monascus purpureus* is one of the most important dual-purpose microbial resources in China and is widely used in many fields, such as food, medical and biocatalytic fields. It is well known that *M. purpureus* can produce various kinds of secondary metabolites, one of which is pigment [1]. Natural *Monascus* pigments (MPs) possess wide applications because of their bioactivities containing antibacterial, antitumor, and antioxidant functions, among others [2,3]. Monacolin K, another beneficial metabolite, can inhibit the 3-hydroxy-3-methylglutaryl coenzyme A reductase effectively. Therefore, it has served as a multi-functional dietary supplement to help lower cholesterol levels [4,5]. Similar to MPs, citrinin is a polyketide compound. However, it has nephrotoxic activities in mammals that once aroused enough panic to restrict the wide usage of MPs [6]. Nowadays, many researches are devoted to reducing citrinin and increasing the number of beneficial metabolites. Media conditions are crucial for the growth of fungi and the production of secondary metabolites. While carbon and nitrogen sources are important factors [7], some studies confirmed that an alkaline environment or an extremely low pH could inhibit citrinin production and promote MPs synthesis [8,9]. Metal ions, such as Fe^2+^, Zn^2+^, Mn^2+^, and so on, also played a key role in MPs accumulations [10]. Up to now, there were only a few reports on the influence of Na^+^ on secondary metabolites biosynthesis in *M. purpureus* [11,12].

Saline conditions, hypersaline in particular, could dramatically inhibit cell growth [13,14]. In fungi, salinity stress has been proven to affect the formation of sclerotia [15]. However, fungi could adapt to hypersaline conditions mainly through the high osmolarity glycerol (HOG) pathway. Additionally, salt stress and response elements related to the HOG pathway play an important role in the biosynthesis of the second metabolism [16,17]. Furthermore, salt stress can induce some secondary metabolites biosynthesis in some fungi, which is one effective strategy for resistance to the stress [18]. Most of literature focused on salt stress in plants because saline-sodic soil was one of the limiting factors of crop yields [19,20]. Nowadays, there are reports that fungi, such as *Arbuscular mycorrhyzae*, salt tolerant *Trichoderma*, and so on, could alleviate salt stress in plants [21,22]. It was confirmed that those fungi enhanced plant salt tolerance by improving the activities of antioxidant enzymes [23]. However, NaCl induced osmotic stress, which is responsible for the oxidative stress caused by reactive oxygen species (ROS) [24]. Previous work suggested that ROS were crucial for lovastatin accumulation in *Aspergillus terreus* [25]. These conclusions provide new ideas for the accumulation of beneficial metabolites or the inhibition of citrinin by NaCl.

In this study, different concentrations of NaCl were added to media to explore the relationship between saline and secondary metabolism biosynthesis. Then, the reason for the changed yield of secondary metabolites was further analyzed by measuring the ROS level, catalase (CAT) activity, superoxide dismutase (SOD) activity and total glutathione (T-GSH). The relative expression levels of the MPs synthesis genes (*pksPT* and up-regulator gene (*pigR*)), the citrinin synthesis genes (*pksCT* and transcription regulator gene (*ctnA*)) [26,27] were also determined under NaCl treatments.

## 2. Results

### 2.1. Effect of NaCl on the Growth and Secondary Metabolites of *M. purpureus*

**Figure 1:**
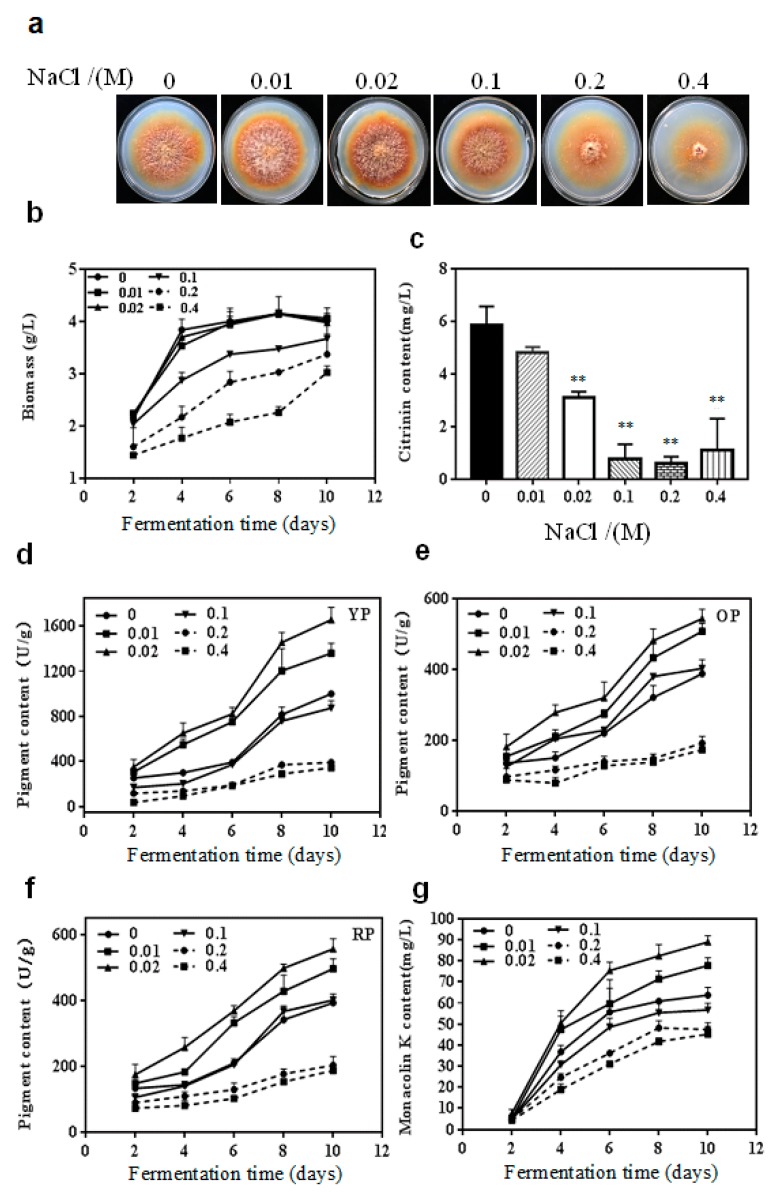
Effect of NaCl on cell growth and secondary metabolites production in *M. purpureus*. (**a**) colony morphology at incubation 10 d; (**b**) biomass during fermentation; (**c**) citrinin content during fermentation 10 d; (**d**) yellow pigment (YP) content during fermentation; (**e**) orange pigment (OP) content during fermentation; (**f**) red pigment (RP) content during fermentation; (**g**) monacolin K content during fermentation. **, *p* < 0.01.

The effect of various concentrations of NaCl (0, 0.01, 0.02, 0.1, 0.2 and 0.4 M) on the growth of *M. purpureus* on potato dextrose agar (PDA) for 10 days is shown in Figure 1a. It could be intuitively observed that the inhibition effect of NaCl on colony size and growth of aerial mycelia was more obvious with increased NaCl concentrations. The cells suspended in potato dextrose broth (PDB) containing different concentrations of NaCl were taken on day 2, 4, 6, 8 and 10 of cultivation to analyze the secondary metabolites production. As presented in Figure 1b, the growth of *M. purpureus* was not obviously affected at any time in the experimental group supplement with 0.01 or 0.02 M NaCl. But the biomass decreased markedly in the experimental group (*p* < 0.01) with the addition of 0.1 M, 0.2 M, and 0.4 M NaCl.

Citrinin was not detected until the 10th day of fermentation (Figure 1c). There was no significant impact on the citrinin content of the experimental group containing 0.01 M NaCl (*p* > 0.05). Nevertheless, NaCl reduced the content of citrinin by 48.0%, 87.2%, 89.7%, and 81.4% at 0.02, 0.1, 0.2, and 0.4 M of NaCl concentrations, respectively, compared with the control.

The yellow, orange and red pigment yields were on an upward trend whether they had NaCl treatment or not. But the production of the three pigments increased slowly at any time during fermentation when 0.2 M and 0.4 M NaCl were added. In contrast, yellow, orange and red pigment contents notably enhanced by 0.01 M and 0.02 M NaCl in the mid and later phases of fermentation. The maximum yellow, orange and red pigment contents were 1655.9, 543.2 and 555.3 U/g during fermentation 10 d, respectively, when cultured with 0.02 M NaCl. The yellow pigment contents decreased during fermentation 4 and 10 d (Figure 1d) but the orange pigment contents markedly increased during fermentation 4 and 8 d (Figure 1e, *p* < 0.05) when treated with 0.1 M NaCl. There was no significant difference in red pigment yields between the 0.1 M NaCl-treated group and the control group (Figure 1f).

NaCl concentrations had no influence on the monacolin K content cultured for 2 days (Figure 1g). With the extension of fermentation time, both 0.01 and 0.02 M NaCl enhanced monacolin K production. A maximum increase of 40% in monacolin K content was observed in the 0.02 M NaCl-treated group in contrast with the control during fermentation 10 d. But the accumulation of monacolin K was dramatically reduced when NaCl concentrations were above 0.1 M.

### 2.2. Effect of NaCl on Antioxidant Enzymes Activities, ROS and T-GSH Contents

**Figure 2:**
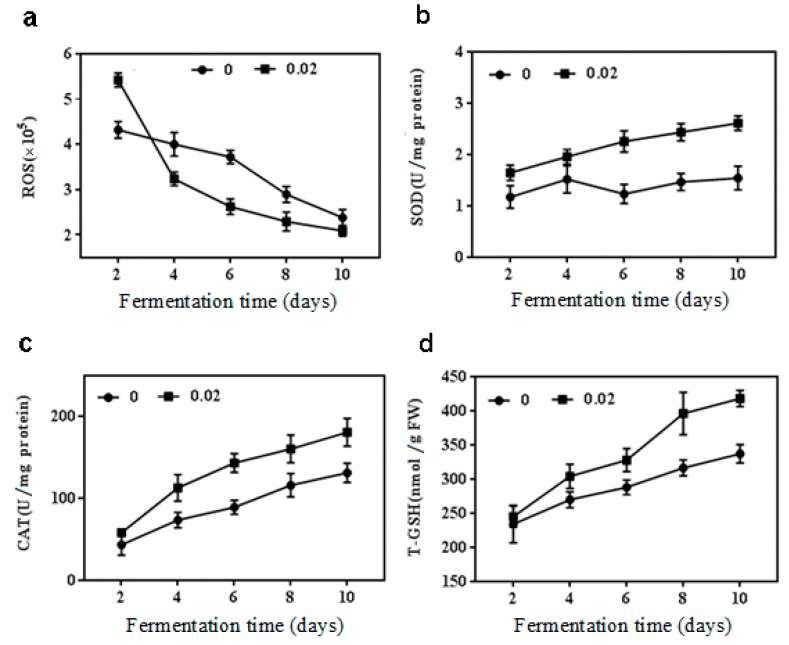
Reactive oxygen species (ROS) content, superoxide dismutase (SOD) and catalase (CAT) activities, total glutathione (T-GSH) production in *M. purpureus* by 0.02 M NaCl treatment. (**a**) ROS content; (**b**) SOD activity; (**c**) CAT activity; (**d**) T-GSH production.

The antioxidant modulation was analyzed to further explore the relationship between NaCl and the secondary metabolism when 0.02 M NaCl was considered as the optimal condition of inhibiting citrinin and stimulating beneficial metabolites without affecting the growth of *M. purpureus*. As shown in Figure 2a, the ROS level had been declining in both treatment and control groups with the extension of the fermentation time. It showed an increase of 26.0% over the ROS level of control during fermentation 2 d with 0.02 M NaCl treatment. But in comparison with the control, the 0.02 M NaCl concentration caused reduction in ROS contents during fermentation 2, 4, 6 and 8 d, and during fermentation 10 d, respectively.

The activities of antioxidant enzymes and the content of T-GSH were observed to show a noticeable ascending trend. The SOD activity was enhanced obviously when the NaCl concentration was at 0.02 M, which raised it 1.3, 1.9, and 1.8 times during fermentation 6, 8 and 10 d, respectively, compared with that of the control group (Figure 2b). CAT activity also increased at 0.02 M NaCl treatment except on day 2 of cultivation (Figure 2c). The effect of 0.02 M NaCl on T-GSH content was presented in Figure 2d. There was no difference in T-GSH content between the treatment group and the control group after fermentation for 2 d. However, the difference became significant with the extension of the fermentation time (*p* < 0.05). Compared with the control, the maximum increase was 43% during fermentation 10 d with 0.02 M NaCl treatment.

### 2.3. Effect of NaCl on Acid and Alkaline Phosphatase Activities

**Figure 3:**
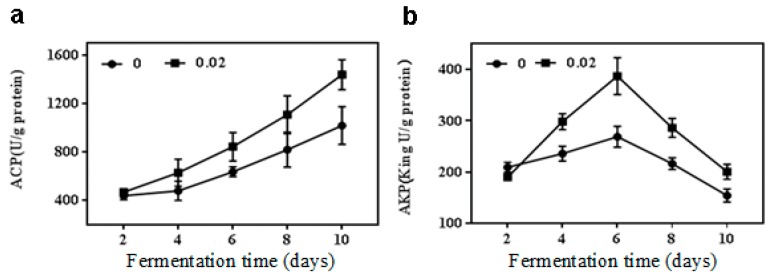
Acid phosphatase (ACP) and alkaline phosphatase (AKP) activities of *M. purpureus* by 0.02 M NaCl treatment. (**a**) ACP activity; (**b**) AKP activity.

The other two indexes (acid and alkaline phosphatase, ACP and AKP activities) were used to further clarify the effect of NaCl on the growth of *M. purpureus* from the aspect of stress regulation. In general, ACP activity was kept at an increase trend as fermentation proceeded regardless of NaCl treatment (Figure 3a). The activity of ACP increased by 28.6% in 0.02 M NaCl treatment compared to the control during fermentation 10 d, and there was no significant difference (*p* > 0.05) between the treatment and the control fermented samples at less than 10 days. However, the activity of AKP increased first and reached the maximum on day 6, and then decreased (Figure 3b). The result showed that 0.02 M NaCl increased the AKP activity by 26.5%, 32.2%, and 30.2% during fermentation 4, 8 and 10 d, respectively, as compared to the control at the same times. The maximum increase was 49.5% on day 6 of fermentation in AKP activity with the treatment group over the control group.

### 2.4. qRT-PCR Analyses of Genes Relative Levels

**Figure 4:**
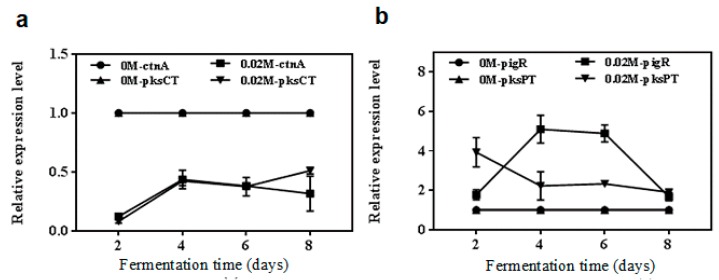
The relative expression levels (RELs) of the *Monascus* pigments (MPs) synthesis genes *pksPT*, *pigR*, citrinin synthesis genes *pksCT* and *ctnA* by qRT-PCR in the 0.02 M NaCl treatment and control groups. (**a**) RELs of *pksPT* and *pigR*; (**b**) RELs of *pksCT* and *ctnA*.

To further clarify the role of NaCl in the biosynthesis processes of metabolites, the transcription levels of the *pksPT*, *pigR*, *pksCT* and *ctnA* genes were analyzed by qRT-PCR in the 0.02 M NaCl treatment and control groups (Figure 4). The relative expression levels (RELs) of *ctnA* and *pksCT*, respectively, in treatment groups both were down-regulated compared with the control (Figure 4a). Moreover, as shown in Figure 4b, the RELs of *pigR* under 0.02 M NaCl concentration showed a trend of being low at the early fermentation stage, and high in the middle period, but remaining low in the later period. However, the RELs of *pksPT* were high first and then decreased under 0.02 M NaCl concentration. The RELs of *pigR* and *pksPT* of 0.02 M NaCl treatment were both higher than those of the control groups during the entire course of fermentation.

## 3. Discussion

It is universally acknowledged that salinity is a serious environmental constraint affecting the growth of most microorganisms [28]. Similar to the findings of Babitha et al. [11], cell growth was significantly inhibited by high salt stress due to reducing water activity [29]. But on the other hand, many secondary metabolites biosynthesis are related to salt stress. For example, marine-derived fungus could produce a new metabolite under high salt stress [30]. As useful metabolites, MPs and monacolin K possess some biological activities and are widely used as potential anticancer and weight-loss drugs [1]. Previous studies have proved that a certain NaCl concentration could increase extracellular red pigment content [11,31]. In this study, the optimum concentration of 0.02 M NaCl was observed to greatly enhance the production of MPs and monacolin K, and to conversely inhibit citrinin biosynthesis. As an antioxidant, the increase of MPs might be a defensive mechanism under salt stress. The phenomenon of a decline in citrinin production and an increase in monacolin K content might be due to the polyketide pathway of metabolites biosynthesis [12,32]. According to the elaboration of Wan et al. [27], NaCl treatment may enhance pigment gene expression, thus resulting in more malonyl-CoA to synthetize monacolin K and less enzymatic condensation of acetyl-CoA with tetraketide to synthetize citrinin. Otherwise, we found that the RELs of both *pksPT* and *pigR* were significantly enhanced, and those of *pksCT* and *ctnA* were apparently reduced vitally in 0.02 M NaCl treatment relative to the control. This result might be due to the molecular mechanism in NaCl that could reduce citrinin content and promote MPs production.

It is worth pointing out that salt stress can lead to metabolic toxicity due to oxidative stress caused by ROS. Fortunately, there is a defense system in organisms to scavenge ROS, which includes antioxidant enzymes and non-enzymes antioxidants, such CAT, SOD, and GSH [33]. In this study, 0.02 M NaCl treatment induced the ROS level to increase during fermentation 2 d, meanwhile there were no changes in the activities of SOD and CAT and in the content of T-GSH, and the three antioxidants were enhanced by salt concentration at the later stage. Thus, it scavenged ROS, showing a similar result to Benmoussa et al. [34]. Besides, the decline of ROS level may partly be ascribed an increase in MPs content. On the other hand, ROS accumulation on day 2 of cultivation could facilitate MPs and monacolin K biosynthesis later. A similar conclusion has been reported in *Aspergillus terreus*, with lovastatin biosynthesis being promoted under ROS accumulation [25].

ACP and AKP activities were also enhanced by 0.02 M NaCl treatment in this study. A similar result was presented in *Trichoderma asperellum* Q1, showing that NaCl could activate ACP and AKP [35]. In addition, the activity of AKP was enhanced by Na^+^ treatment in *Aspergillus caespitosus* [36]. It has been reported that ACP and AKP play key roles in protein dephosphorylation. And protein phosphorylation and dephosphorylation were the most important ways of regulating cell metabolism [37,38]. The ACP and AKP might play a role in the cell growth and morphology of fungi [39]. We proposed that the reduction of mycelia caused by NaCl treatment may also be ascribed to the increase of ACP and AKP activities, which coincided with the finding of Reyes et al. [40].

This study proved that NaCl could inhibit the biosynthesis of citrinin and contribute to the production of beneficial secondary metabolites (MPs and monacolin K). The reason behind how the metabolites productions were affected was explained through the ROS levels and the RELs of genes of citrinin and MPs.

## 4. Materials and Methods

### 4.1. Fungal Strain, Culture Media, and Growth Conditions

*Monascus purpureus* SKY219 (Microbiology Laboratory of Yangtze University, Jingzhou, China) was maintained on potato dextrose agar (PDA) slants at 4 °C. The strain was cultivated on Czapek yeast extract ager (CYA) slants for sporulation at 30 °C for 10 d. Spores were harvested by 10 mL sterile water and inoculated to analyze growth and secondary metabolites.

For observing the difference of colony morphology, spore suspension was concentrated and inoculated into 25 mL of PDA and incubated at 30 °C. Fifty mL of potato dextrose broth (PDB) with 2 × 10^3^ spores per mL was fermented in a 100 mL triangular flask at 30 °C in a rotary shaker (at 200 rpm) and used for the secondary metabolites production and the total RNA extraction. Cultivated fermentation liquor was collected during fermentation 2, 4, 6, 8, and 10 d, respectively. It was used to measure citrinin, MPs, monacolin K, ROS, T-GSH content, and CAT, SOD, ACP, and AKP activities.

An aqueous solution of NaCl was sterilized by filtering through 0.22-μm polyvinylidenedifluoride syringe filters (Millipore Ltd., Bedford, MA, USA). NaCl solutions were supplemented into PDA to observe phenotypic characterization at the 10th day of cultivation and into PDB medium to measure various indicators at different times with final concentrations of 0.01, 0.02, 0.1, 0.2 and 0.4 M, respectively. 

### 4.2. Biomass Determination

Sediments were collected after fermentation by centrifugation of the fermentation broth (5000 rpm, 10 min, 4 °C). Then the sediments were washed three times and dried to constant weight at 60 °C. Thus, the biomass (g/L) was obtained as dry cell weight per unit volume of fermented broth.

### 4.3. Citrinin, Monacolin K and MPs Determination

Citrinin, monacolin K and MPs contents were measured according to the method of Wan et al. with a minor modification [27].

Both monacolin K and citrinin concentrations were estimated by using high performance liquid chromatography (HPLC). Citrinin and monacolin K standard compounds from ShanghaiYuanye (ShanghaiYuanye Bio-Technology Co., Ltd, Shanghai, China) were used for confirmation of HPLC analysis to construct a standard curve. Citrinin was extracted from 1 mL of the filtrate using an equal volume of toluene-ethyl acetate-formic acid (7:3:1, *v*/*v*/*v*). The elution was monitored using a fluorescence detector at 500 nm emission wavelength and 330 nm excitation wavelength. To detect the monacolin K content, the method of extraction and HPLC was the same with Wan et al. [27].

Pigments in mycelia were analyzed by ultraviolet (UV) spectrophotometer (UV2600, Shimadzu, Kyoto, Japan). Cells were disrupted by sonification (120 W, 40 min) and extracted by 70% (*v*/*v*) ethanol at 60 °C for 1 h. Three kinds of MPs, the yellow, orange and red pigments, were determined by measuring the absorbance of supernatant after vacuum filtration at 410 nm, 465 nm, and 500 nm, respectively. The pigments were quantified by calculating absorbance and dilution and presented as absorbance units (U/g) per gram of dry mycelia.

### 4.4. ROS Determination

According to the method of Miranda et al. [25], ROS were quantified through Fluorogenic probe H_2_DCF-DA. Ten milligrams of mycelium obtained at various periods of growth was incubated for 0.5 h at 4 °C in the dark with 1 mL of H_2_DCF-DA (20 μM) solution and filtered. One milliliter of cold PBS buffer was added to a 1.5 mL black microtube containing 500 μg mycelium powder grounded through liquid nitrogen and mixed. After being centrifuged (12,000× *g*, 15 min, 4 °C), 200 μL of supernatant was analyzed using an enzyme labeling instrument (λ_ex_ = 488 nm, λ_em_ = 530 nm).

### 4.5. SOD, CAT Activities and T-GSH Determination

The activities of SOD, CAT, and the content T-GSH were assayed according to the instructions of the factory kit (Shanghai Beyotime Biotechnology, Shanghai, China). The SOD assay kit (catalog number: S0109) described the SOD activity (U/mg protein) as the quantity of protein inhibiting 50% photoreduction of nitroblue tetrazolium (NBT), and this was measured at 560 nm. CAT activity was estimated referring to the kit (catalog number: S0051), and the decline in the absorbance of hydrogen peroxide was measured at 520 nm. One unit of CAT equaled to the amount of enzyme that could catalyze the destruction of 1 μM H_2_O_2_ within 60 s at 25 °C and pH 7.0. The amount of yellow color produced by 5,5’-Dithiobis-(2-nitrobenzoic acid) (DTNB) directly reflected the content of T-GSH reacting for 25 min at 25 °C, according to the assay kit (catalog number: S0052). It was measured by the absorbance at 412 nm.

### 4.6. Acid and Alkaline Phosphatase Activities Determination

The procedure of Gianinazzi-Pearson et al. [41] was employed for the estimation of acid and alkaline phosphatase (ACP and AKP) activities. The wavelength was taken at 410 nm. One unit of ACP activity was described as 1 mg phenol reduced by per g protein in tissue within 30 min at 37 °C. AKP enzyme activity (King unit/g) was expressed as 1 mg phenol released from per g protein within 15 min at 37 °C.

### 4.7. Quantitative RT-PCR Analyses

The quantitative RT-PCR (qRT-PCR) analysis was carried out according to the method of Wan et al. [27]. In brief, the total RNA from hyphae was extracted by a Trizol reagent. The primers sequences, designed based on DNA sequences (https://genome.jgi.doe.gov/Monpu1/Monpu1.home.html) from which the introns had been removed, are shown in Table 1. RELs were examined by a Toyobo Thunderbird SYBR qRT-PCR Mix kit (Toyobo, Osaka, Japan) and GAPDH was used as the reference gene.

### 4.8. Statistical Analyses

Data are presented as mean ± standard deviations (SD) from three independent experiments measured in triplicate. Statistically significant differences were analyzed by the Tukey’s multiple-comparison procedure test or by one-way analysis of variance (ANOVA). Statistical significance was determined at the level *p* < 0.05. The analyses were performed using SPSS version 12.0 (Statistical Package for Social Sciences, SPSS Inc., Chicago, IL, USA).

## Figures and Tables

**Table 1:** Primers used in this study.

Name	Sequences(5’→3’)	Descriptions
*ctnA-S*	AACCATGGAGGCATTGGACC	For qRT-PCR analysis of *ctnA*
*ctnA*-A	CCTTGTCGGTCACACCGAAT	
*pksCT*-S	TTCTGACACGACCATCAGCC	For qRT-PCR analysis of *pksCT*
*pksCT*-A	ACGACGACGAGTGTCAGTTC	
*pksPT*-S	GGCAACCTTCAGTCCGCTAT	For qRT-PCR analysis of *pksPT*
*pksPT*-A	GATCAGTGCGATGCCATGTG	
*pigR*-S	ACTCTGGAAAGCTGCTTCGG	For qRT-PCR analysis of *pigR*
*pigR*-A	GGACGTTCTGGATGGCGTAT	
*GAPDH*-S	CAAGCTCACTGGCATGTCTATG	For qRT-PCR analysis of *GAPDH*
*GAPDH*-A	AAGTTCGAGTTGAGGGCGATA	
