# Correction: Zhen, Z. et al. NaCl Inhibits Citrinin and Stimulates *Monascus* Pigments and Monacolin K Production. *Toxins* 2019, *11*, 118

**DOI:** 10.3390/toxins11040183

**Published:** 2019-03-27

**Authors:** Zhixin Zhen, Xiaoqian Xiong, Yingbao Liu, Jialan Zhang, Shaojin Wang, Li Li, Mengxiang Gao

**Affiliations:** College of Life Science, Yangtze University, Jingzhou 434025, China; zhenzhixin@yangtzeu.edu.cn (Z.Z.); 201672408@yangtzeu.edu.cn (X.X.); liuyingbao@yangtzeu.edu.cn (Y.L.); zhangjl@yangtzeu.edu.cn (J.Z.); shaojinwang@nwafu.edu.cn (S.W.); lily2012@yangtzeu.edu.cn (L.L.)

The authors wish to make the following correction to their paper [1]:

In the Abstract section, the fifth sentence should be replaced with, “It was verified through the transcriptional down-regulation of citrinin synthesis genes (*pksCT* and *ctnA*) and up-regulation of the *Monascus* pigments (MPs) synthesis genes (*pksPT* and *pigR*).”

We apologize for any inconvenience caused to readers of *Toxins* by this change.
